# Predicting IVF outcome in poor ovarian responders

**DOI:** 10.1186/s12905-022-01964-y

**Published:** 2022-09-30

**Authors:** Oshrit Lebovitz, Jigal Haas, Nitzan Mor, Eran Zilberberg, Adva Aizer, Michal Kirshenbaum, Raoul Orvieto, Ravit Nahum

**Affiliations:** 1 IVF Unit, Department of Obstetrics and Gynecology, Chaim Sheba Medical Center, 52621 Tel- Hashomer, Israel; 2 Sackler Faculty of Medicine, Tel Aviv University, 69978 Tel Aviv, Israel

**Keywords:** In vitro fertilization, Poor ovarian response, Live birth, Bologna criteria

## Abstract

**Background:**

Poor responders to ovarian stimulation are one of the most challenging populations to treat. As a failed cycle can cause a considerable emotional and economical loss, adequate fertility counseling addressing patients’ expectations are highly important when facing patients with poor ovarian response. The study aimed to evaluate reproductive outcomes and to identify factors associated with live birth (LB) after fresh autologous IVF/intracytoplasmic sperm injection (ICSI) cycles of patients fulfilling the Bologna criteria for poor ovarian response (POR).

**Methods:**

A retrospective study included 751 IVF/ICSI treatment cycles which yielded up to three retrieved oocytes, at a tertiary referral hospital between January 2016 and February 2020. A logistic regression analysis was used to adjust for confounders.

**Results:**

Clinical pregnancy and LB rate per cycle were significantly higher among women younger versus older than 40 years (9.8% and 6.8% vs 4.5% and 2.1%, *p* < 0.01, respectively). Patients who achieved LB were significantly younger, had higher number of oocytes retrieved, fertilization rate and top-quality embryos (*p* < 0.05). Multivariable regression analysis identified patient’s age (OR 0.90; 95% CI 0.845–0.97; *p* = 0.005) and mean number retrieved oocytes (OR 1.95; 95% CI 1.20–3.16; *p* = 0.007) as factors significantly associated with the probability of a LB.

**Conclusions:**

The woman’s age and the number of retrieved oocytes are both independent predicting factors of live birth in poor ovarian responders. Considering the risks, the high financial investment and poor reproductive outcomes involved in IVF treatments, raises questions regarding the adequacy of providing treatments in these patients' population. POR younger than 40 years may represent a possible exception due to acceptable probability for a LB.

## Introduction

Ovarian stimulation (OS) plays a central role in the success of in vitro fertilization (IVF) cycles. The goal of OS is to recruit and develop multiple dominant follicles. This may lead to the retrieval of many oocytes which allows many potential embryos for transfer [1]. Overall, between 10 and15 oocytes are considered to be the optimal ovarian response following OS [2]. Despite great advances in assisted reproductive technology (ART), treating patients with poor ovarian response (POR) is still considered a major challenge in reproductive medicine. POR patients are less likely to conceive and moreover, will have higher risk of cycle cancellation. The definition of POR has been standardized in the Bologna criteria established by the European Society of Human Reproduction and Embryology (ESHRE) in 2011 [3].

Due to the heterogeneous risk factors for POR, there is a wide variability in the incidence, estimated to be between 9 and 24% of patients undergoing OS for IVF [4, 5]. However, with the persistent and pronounced trend towards delayed childbearing across the western world, the incidence may be slightly higher.

Several OS protocols have been proposed for improving the ovarian response of poor responders [6–8], yet none have demonstrated their superiority. Most of the cycle treatments end with poor outcome of fewer pregnancies and livebirth rate. Overall, poor responders require longer stimulation duration, associated with high doses of gonadotropins and higher cost. Still cycle cancellation rates are increased [9]

A cycle cancellation due to a failure of an oocyte retrieval or an available embryo to transfer is a very stressful event, given the significant emotional and financial investment involved in IVF treatment. Therefore, adequate fertility counselling addressing patients’ expectations, as well as adjusting the appropriate treatment strategy, is highly important when facing patient with POR.

Most countries do not provide insurance coverage for IVF treatments, and even those who do, offer only partial financial coverage limiting the number of treatments provided. Consequently, the financial factor significantly hinders the number of cycles that can be carried out. The situation in Israel is unique as the national health insurance provides financial coverage for repeated IVF cycles for the first two children, or up to the age of 45 years, usually regardless of the ovarian reserve.

Prompted by the aforementioned circumstances, we aimed to evaluate IVF cycle outcomes of patients with “genuine” POR defined according to the Bologna criteria and who had up to three retrieved oocytes in response to conventional control ovarian hyperstimulation, in a cost-free environment and with no restriction on the number of IVF treatment cycles. Furthermore, we sought to identify factors that are associated with a live birth in patients with POR. This may assist counselling by fertility specialists and the expectations of patients in adjusting the appropriate treatment strategy of POR patients. Our results might be of interest especially in countries in which egg donation is prohibited, or when multiple repeated IVF cycles attempts are financially affordable.

## Patients and methods

This was a historical cohort study which evaluated all consecutive medical records of infertile women who attended the Sheba Medical Center IVF unit for IVF cycle attempt from January 2016 through February 2020. Inclusion criteria were infertile women aged 18 to younger than 45, fulfilling the Bologna criteria for POR [3], who underwent OS using autologous oocytes and yielded up to three oocytes. Excluded were patients with azoospermia or severe male factor, preimplantation genetic testing, women undergoing fertility preservation (freezing oocytes/ embryos), and patients using surrogacy.

Basic clinical characteristics, infertility treatment related variables and cycle outcomes were abstracted from the patients’ medical records.

The study was approved by the Institutional Review Board (IRB) at Sheba Medical Center. Informed consent was not required.

Ovarian stimulation was performed using one of the following protocols: the multiple-dose GnRH antagonist, the long GnRH agonist (GnRH-a) suppressive, the short GnRH agonist (Flare), and the modified natural cycle-IVF (MNC-IVF) protocol. The selection of OS protocol used, and gonadotrophin dose were decided by the treating physician. In all protocol’s gonadotrophin doses were administrated in variable doses (with a minimal daily dose of 300 IU), and further adjusted based on ultrasound scan and serum estradiol (E2) levels obtained every 2–3 days. Final follicular maturation was induced with one of the two trigger modes: (i) hCG (250 mcg Ovitrelle, Merck) 36 h before oocyte retrieval, (ii) hCG (250 mcg) and GnRH- agonist (Decapeptyl 0.2 mg), 36 h prior to oocyte retrieval, respectively (dual trigger), based on treating physician preference [10]. The trigger day was based on the lead follicular cohort, usually when leading follicle measures > 17 mm for maximal size. A transvaginal, ultrasound-guided follicular aspiration was conducted 36 h after triggering administration. Retrieved oocytes were, according to sperm quality, either inseminated by conventional IVF or by intracytoplasmic sperm injection (ICSI), as previously described [11]. Fertilization was determined by the presence of two pronuclei (2PN) and two polar bodies. Each embryo was cultured separately and evaluated after 48/72 h. Cleavage embryos were defined as top quality embryos (TQE) if they had four to five blastomeres cells on day 2 and/ or 7 or 8 blastomeres on day 3, equally- sized blastomeres, contained < 15% fragmentation, and exhibited no apparent morphological abnormality [12]. Poor quality embryos consisted of all other embryos.

Luteal support was initiated one day after oocyte pick up and consist of vaginal, P.O or I.M progesterone. In general, embryo transfer took place 2–3 days after oocyte retrieval. The remaining viable embryos were cryopreserved.

Pregnancies were confirmed with beta-hCG levels, typically measured 14 days after embryo transfer. A Clinical pregnancy was confirmed when a gestational sac with fetal heartbeat was visible on ultrasound examination 6 weeks after embryo transfer. Delivery of a live infant was defined as any birth event where one or more live babies were born at ≥ 24 weeks’ gestation.

## Statistical analysis

Statistical analysis was performed using the SPSS software package version 25 (SPSS Inc., Chicago, IL). Continuous variables were presented as means and standard deviation (SD). Categorical variables were presented as numbers and percentages. Differences in variables were statistically analyzed by Student’s t-test, Fisher exact test, and Pearson chi-square test, as appropriate. Normality of variables was assessed via Shapiro-Wilks test of normality.

To further investigate predictors for a live birth, a multivariate logistic regression analysis was used controlling for confounding effects that included the patients’ age, protocol type, stimulation duration, peak E2 level, number of dominant follicles at trigger day, and number of retrieved oocytes.

Two-sided *p*-values of < 0.05 were accepted as statistically significant.

## Results

During the study period, 528 patients met the inclusion criteria. The analysis included the outcomes of 751 fresh IVF/ ICSI cycles which yielded up to three oocytes. Of these, 265 cycles occurred among women $\le$ 40 years, and 486 cycles in women > 40 years. Baseline characteristics, IVF cycle outcomes and final reproductive outcomes stratified according to patient’s age are presented in Table 1.

**Table 1:** Patients’ characteristics and cycle outcomes by woman’s age group

	< 40Y	> 40Y	*p*
Number of cycles	265	486	
Age (years), mean ± SD	35.81 ± 3.86	42.70 ± 1.42	< 0.001
BMI (kg/m^2^), mean ± SD	25.64 ± 6.11	26.31 ± 6.19	0.1789
Gravity, mean ± SD	1.15 ± 1.49	1.27 ± 1.53	0.2991
Parity, mean ± SD	0.44 ± 0.78	0.46 ± 0.64	0.7150
Protocol			0.0391
Antagonist, *n* (%)	184 (69.4%)	352 (72.4%)	
MNC, *n* (%)	67 (25.3%)	88 (18.1%)	
Short agonist, *n* (%)	9 (3.4%)	27 (5.6%)	
Long agonist, *n* (%)	5 (1.9%)	19 (3.9%)	
Day 3 FSH levels (IU/L), mean ± SD	12.97 ± 8.97	13.60 ± 11.38	0.5261
Duration of stimulation (days), mean ± SD	8.58 ± 3.76	9.42 ± 3.65	0.003
Total dose of gonadotropins (IU), mean ± SD	3307.3 ± 2245.6	4332.3 ± 2409.9	< 0.001
No. of follicles ≥ 15 mm, mean ± SD	1.68 ± 0.76	1.66 ± 0.74	0.7976
Leading follicle size (mm), mean ± SD	18.53 ± 2.17	18.10 ± 2.04	0.006
E_2_ level at the trigger day (pmol/L), mean ± SD	1750.3 ± 1115.5	1769.4 ± 939.6	0.802
Trigger			0.403
hCG, *n* (%)	192 (72.5%)	338 (69.6%)	
Dual trigger, *n* (%)	73 (27.5%)	148 (30.4%)	
Progesterone levels at the trigger day (nmol/L), mean ± SD	1.43 ± 1.04	1.50 ± 1.21	0.435
No. of retrieved oocytes, mean ± SD	1.55 ± 0.99	1.54 ± 0.97	0.9217
Cycles with no oocytes, *n* (%)	41 (15.47%)	78 (16.05%)	0.852
Top quality embryos, mean ± SD	0.37 ± 0.59	0.33 ± 0.55	0.391
Fertilization rate, mean ± SD	0.63 ± 0.40	0.62 ± 0.4	0.6315
Pregnancy rate per cycle, *n* (%)	26/265(9.81%)	22/486 (4.53%)	0.004
Live birth per cycle, *n* (%)	18/265 (6.79%)	10/486 (2.06%)	0.001

*BMI*, body mass index, *MNC*, modified natural cycle

Baseline characteristics among patients younger versus older than 40 years were similar regarding gravidity, parity, body mass index (BMI) and basal FSH level. Duration of OS and the total gonadotrophins dose used were significantly higher in women older than 40 years (9.42 ± 3.65 days, and 4332.3 ± 2409.9 IU vs 8.58 ± 3.76 days and 3307.3 ± 2245.6 IU, respectively, *p* < 0.01).

During the study period, 48 (9.1%) women conceived, and 28 (5.3%) gave birth to a live infant.

Pregnancy rate and live birth rate per cycle were significantly higher among patients $\le$ 40-years-old, as compared to patients above 40 years (9.8% and 6.8% versus 4.5% and 2.1%, *p* < 0.01, respectively; Table 1).

A further analysis comparing cycles resulted in live birth versus those which did not, is presented in Table 2. The live birth group was statistically significantly younger (mean ± SD, 37.65 ± 4.81 years. vs 40.37 ± 4.12 years., *p* < 0.001), had more oocytes retrieved (2.14 ± 0.79 vs 1.53 ± 0.98; *p* = 0.001), higher fertilization rate (0.80 ± 0.23 vs 0.51 ± 0.43; *p* < 0.001), and greater number of TQEs (0.65 ± 0.55 vs 0.34 ± 0.58; *p* < 0.01) compared with the group who did not achieve live birth.

**Table 2:** Comparison between cycles resulted in live birth versus no live birth

	Live birth (*n* = 28)	No live birth (*n* = 723)	*p*
Age (years), mean ± SD	37.65 ± 4.81	40.37 ± 4.12	0.0006
BMI (kg/m^2^), mean ± SD	25.13 ± 6.23	26.09 ± 6.16	0.467
Protocol			0.147
Antagonist, *n* (%)	26 (92.9%)	510 (70.5%)	
MNC, *n* (%)	2 (7.1%)	153 (21.2%)	
Short agonist, *n* (%)	0 (0%)	36 (5.0%)	
Long agonist, *n* (%)	0 (0%)	24 (3.3%)	
Day-3 FSH (IU/L), mean ± SD	12.05 ± 5.64	13.54 ± 10.82	0.552
Duration of stimulation (days), mean ± SD	8.97 ± 2.63	9.12 ± 3.75	0.83
Total dose of gonadotropins (IU), mean ± SD	3310.33 ± 1720.23	3987.13 ± 2420.71	0.136
No. of follicles ≥ 15 mm, mean ± SD	1.79 ± 0.77	1.66 ± 0.75	0.364
Leading follicle size (mm), mean ± SD	18.69 ± 1.95	18.24 ± 2.10	0.254
E_2_ level at the trigger day (pmol/L), mean ± SD	1873.62 ± 806.21	1756.7 ± 1010.74	0.538
Trigger			0.542
hCG, *n* (%)	19 (65.5%)	511 (70.8%)	
Dual trigger, *n* (%)	10 (34.5%)	211 (29.2%)	
No. of retrieved oocytes, mean ± SD	2.14 ± 0.79	1.53 ± 0.98	0.001
Fertilization rate, mean ± SD	0.80 ± 0.23	0.51 ± 0.43	0.0004
Top quality embryos, mean ± SD	0.65 ± 0.55	0.34 ± 0.58	0.004
No. of embryos transferred			< 0.001
0, *n* (%)			
1, *n* (%)	–	341 (47.2%)	
2, *n* (%)	17 (60.7%)	258 (35.7%)	
3, *n* (%)	11 (39.3%)	114 (15.8%)	
	–	10 (1.4%)	

*BMI*, body mass index, *MNC*, modified natural cycle

When controlling for potential covariates in a multivariable regression analysis, however, the only statistically significant factors associated with achieving a live birth were patient's age (OR 0.90; 95% CI 0.845–0.97; *p* = 0.005) and mean number retrieved oocytes (OR 1.95; 95% CI 1.20–3.16; *p* = 0.007) (Table 3).

**Table 3:** Multivariable regression analysis for factors associated with live birth in POR patient’s undergoing IVF

Predictor	Odds ratio	95% CI	*p* value
Age (years)	0.905	0.84–0.97	0.005
Type of protocol			NS
Antagonist	Reference		
MNC	0.31	0.07–1.39	NS
Short agonist	0	0	NS
Long agonist	0	0	NS
Stimulation duration (days)	0.91	0.78–1.06	NS
No. of follicles ≥ 15 mm	0.96	0.53–1.75	NS
E_2_ level at the trigger day (pmol/L)	0.96	0.60–1.55	NS
No. of retrieved oocytes	1.95	1.21–3.16	0.007

*MNC*, modified natural cycle, *NS*, not significant

## Discussion

The present study was designed to provide women with genuine POR according to the Bologna criteria with prognostic information regarding IVF cycles outcomes and their chances of a live birth. Our data demonstrates that the probabilities of POR patients achieving a live birth are best with younger age and higher number of retrieved oocytes.

The age-related decline in ovarian reserve in women over 40 years is well documented [13, 14]. Nevertheless, for some women the physiologic decrease in ovarian function occurs earlier. The subgroup of patients termed “poor ovarian response” represents the most challenging group of patients to treat, as this population are at high risk for inadequate response during IVF treatment and lower pregnancy and live birth rates [15].

Several pretreatment diagnostic tests including basal FSH, AFC, inhibin B, and AMH have been used for prognosticating a poor responder to stimulation. More accurate prognostic information can be derived by the completion of the ovarian stimulation cycle, as this “stress test” explores the capacity of the ovary to produce enough oocytes.

Previous studies found that the recurrence rate of poor ovarian response during the subsequent ovarian stimulation was 54–62% [16, 17].

Dilemmas that physicians may encounter while treating patients with POR may arise from the tendency to include this subgroup of patients as a homogenous group and therefore labeling similar treatment and prognosis to all patients. In fact, this is a heterogeneous group composed of different ages and various causes which led to POR. Thus, the question arises whether or not there are any prognostic factors that will mark some of the poor responders' patients with an acceptable prognosis for live birth following IVF treatment. Identifying such prognostic factors will assist fertility providers in counseling POR patients as to whether it is useful or not to start and/or to continue with IVF treatment. Moreover, since the cost of IVF remains the most significant barrier to infertility care and while attempts to reduce drugs cost (by using biosimilar products) were not shown to be cost-effectiveness [18], it is, therefore, necessary to constantly assess treatment outcomes, especially when treating women with POR where the cost of the treatments and drugs expected to be even higher.

A woman’s age is considered one of the most significant single determinants affecting chances of conception, either naturally or via ART [19–21]. Therefore, while counseling patients regarding their prognosis, age should be strongly considered. Hanoch et al. [22] conducted a study in which they evaluated differences in pregnancy rates of young versus older low responder patients. They reported significantly higher clinical pregnancy rate among young (20–30 years.) low responder patients (19.3% vs 6.5%). Accordingly, they concluded that young age protects from the deleterious effect of POR. In a literature review, Oudendijk et al. [15] concluded that older poor responders have lower pregnancy rates compared with younger poor responders (1.5–12.7% vs 13–35%).

The findings of the present study are in line with the above-mentioned studies. Woman’s age was found to be negatively associated with the probability of achieving a live birth in lower responder’s patient population.

The distinct effect of female’s age on reproductive outcome is explained by the declining oocytes quality, which coincides with a progressive decrease in the primordial follicle number that occurs with female’s aging [23–25].

The age-induced oocyte quality impairment is closely associated with chromosomal abnormalities, and mitochondrial dysfunction [26, 27]. Women of older age are subjected to a higher number of aneuploid embryos which are more likely to arrest in extended culture [28]. This probably explains the substantially lower live birth rate of women of advanced maternal age.

The primordial follicle pool depletion is common not merely in women of advanced maternal age, but also in most poor ovarian responders, irrespective of age [29]. This significantly limits the success of assisted reproduction treatment [30].

Since both groups of women, advanced maternal age (AMA) and poor ovarian responders, as a subgroup, share the same physiologic follicular pool depletion, the question that arises is whether young poor responders also exhibit a reduction in oocyte quality like AMA women (i.e., high risk of aneuploidy, poor embryo development).

In an attempt to address this question, a recent retrospective study published by Morin et al. [31] found that compared to normal responders, a fertilized oocyte retrieved from a young poor responder patient (< 38 years) is no less likely to form a quality blastocyst, be euploid or produce a live birth. They concluded that an oocyte retrieved from a poor responder patient performs similarly to that from age-matched controls.

The present study demonstrates a significant higher live birth rate in poor ovarian response who are younger than 40 years compared to poor responders > 40 years (7% vs 2%, respectively). This finding as well as the results of the multivariate analysis strongly suggest that a woman’s chronological age is a critical factor which affects IVF outcome and protects against the adverse effect of poor ovarian response.

Another factor which the present study found to be of relevance in determining the prospects of Bologna poor responders for a live birth is the number of oocytes retrieved.

Although the number of oocytes per se is not an indicator of their quality, yet the lower the degree of poor ovarian response, there will be more oocytes to retrieve and subsequently more embryos to transfer, which may improve the chances for pregnancy.

The results of the multivariate analysis which found the number of oocytes retrieved and not the number of dominant follicles visualized ultrasonographically as an independent predictor of live birth could be explained by the fact that oocytes could not be retrieved from some follicles during ovum pick up. The fact that this index cannot be determined in advance limits its value in counseling and preparing POR patients before embarking on IVF treatment.

Our findings are in agreement with previous studies which found positive association between the number of oocytes retrieved and pregnancy/live birth rates in poor responders [15, 32].

As a factor in informed decision-making and as an integral part of proper reproductive counseling, infertile poor ovarian responders should be aware of alternative ways to achieve successful reproduction. Using ovum donation comprises a good alternative for this POR population. The substantial benefit lies in the greater chances of live birth. Several countries, however, prohibit the use of an ovum donation. In such countries, the information from our study might be of particular interest, as the results show a fair chance of attaining a live birth in patients with POR who are younger than 40 years.

The present study contributes to research dealing with IVF outcomes in poor responder patients. It is important to stress that when reading through the current published literature there is a lack of uniformity regarding the definition of poor responders (from fewer than two up to five dominant follicles) [33], which explains the differences in the studies’ results. To the best of our knowledge, the current study is among the largest experiences of poor responders, defined according to the Bologna criteria, who yielded up to 3 retrieved oocytes.

The unique health care system in which this study was conducted provides financial coverage for repeated IVF cycles. The absence of financial constraints enables patients to complete a higher number of IVF cycles when necessary and according to their desire and therefore provides information on an increased number of treatment cycles, which in other places might have been canceled due to poor response.

The inclusion of a high number of treatment cycles strengthens the power of the study results. It offers a more realistic and accurate assessment of the markers predicting success in the poor ovarian response population, thus contributing valuable information for fertility providers and patients alike. The study results can contribute to the quality of counseling before starting treatments and can assist physicians in determining the best candidate for treatment among poor ovarian responders. These would be especially important in countries where egg donation is prohibited, or multiple repeated IVF cycle attempts are financially affordable.

The limitations of the study should also be noted. First, as this was a retrospective study, it had the inherent biases that might affect the results. Although we tried to minimize selection bias by using rigorous inclusion criteria, we cannot exclude unknown confounders that might have affected the results. In addition, AMH values could not be analysed as they are not part of the local routine testing. Of notice, FSH which is considered a reliable test marker to predict ovarian response to stimulation, according to our results was found to have no predictive value on treatment outcome of poor responders.

Improving the cycle outcome is one of the goals of fertility providers.

However, taking into consideration the balance between risks, costs (financial and emotional) and benefits involved in providing IVF treatment for POR, the question emerges is whether additional cycles using the women’s autologous oocytes are justified or continuation of treatment should be discouraged.

Establishing an expected live birth rate along multiple IVF cycle treatments is essential in determining the benefit to women with POR. Given the present study data, it appears that continuation with IVF treatments seems reasonable in patients younger than 40 years, since in these poor responder’s pregnancy rates were 10%, and live birth rates were 7% per cycle. Yet, for poor responders’ women $>$ 40 there appeared to be hardly any justification for any further IVF treatment, due to very poor outcome (4.5% pregnancy rate, and 2% live birth rate per cycle).

In conclusion, the woman’s age and the number of retrieved oocytes are both independent predicting factors of live birth in poor ovarian responders according to the Bologna criteria.

All poor ovarian responders should be informed regarding the meager probability of achieving a live birth. Specifically, POR women $>$ 40 years should be encouraged to apply for the process of egg donation, which will improve their chances of a live birth substantially.

## Data Availability

The datasets used or analyzed during the current study are available from the corresponding author on reasonable request.

## Abbreviations

IVF: In vitro fertilization
ICSI: Intracytoplasmic sperm injection
LB: Live birth
POR: Poor ovarian response
OS: Ovarian stimulation
ART: Assisted reproductive technology
GnRH-a: Gonadotropin-releasing hormone agonist
MNC: Modified natural cycle
TQE: Top quality embryo
E2: Estradiol
BMI: Body mass index
AMA: Advanced maternal age
